# Hyperfunctional Neutrophils in Aged Mice Are Linked to Enhanced Bone Loss in Ligature-Induced Periodontitis

**DOI:** 10.3390/dj13060244

**Published:** 2025-05-29

**Authors:** Antoine Magne, Chunxiang Sun, Sina Zargaran, Jeffrey W. Chadwick, Abdelahhad Barbour, Michael Glogauer

**Affiliations:** 1 Faculty of Dentistry, University of Toronto, Toronto, ON M5G 1G6, Canada; antoine.magne@univ-tlse3.fr (A.M.); chunxiang.sun@dentistry.utoronto.ca (C.S.); siina.zargaran@mail.utoronto.ca (S.Z.); abdelahhad.barbour@utoronto.ca (A.B.); 2 Faculty of Science and Engineering, University of Toulouse, 31400 Toulouse, France; 3 University of Texas Health Science Center, Houston, TX 77030, USA; jeffrey.w.chadwick@uth.tmc.edu; 4 Department of Dental Oncology, Maxillofacial and Ocular Prosthetics, Princess Margaret Cancer Centre, Toronto, ON M5G 2M9, Canada

**Keywords:** aging, neutrophils, periodontal disease, animal model

## Abstract

**Background/Objectives:** Aging alters neutrophil functions, which may contribute to the progression and severity of periodontitis-related alveolar bone loss. Neutrophils play a key role in immune defense. However, the effects of aging on neutrophil functions and their contribution to periodontal disease remain unclear. This study examined age-related neutrophil dysfunction and its impact on periodontal bone loss. **Methods**: We used young (6 weeks old) and aged (18 months old) C57BL/6 mice to assess age-related neutrophil function. Neutrophil migration, superoxide production, phagocytic activity, and NETosis were evaluated. A peritonitis model and a ligature-induced periodontitis model were employed to investigate the relationship between neutrophil activity and alveolar bone loss. **Results**: Neutrophils from aged mice exhibited reduced migration toward pathogens compared to those from young mice. However, aged neutrophils showed increased superoxide production, elevated phagocytic activity, and enhanced NETosis. In the periodontitis models, these age-related neutrophil alterations coincided with accelerated alveolar bone loss in aged mice. **Conclusions**: The findings indicate that aging is linked to dysregulated neutrophil functions, characterized by excessive oxidative stress, heightened phagocytosis, and increased NETosis. These functional changes may contribute to immune dysregulation and tissue damage, thereby promoting age-related alveolar bone loss in periodontitis.

## 1. Introduction

Neutrophils are essential components of the innate immune system and are among the first responders to infection [1]. They act by rapidly recognizing and migrating to sites of infection, where they neutralize pathogens through several mechanisms [2]. These include phagocytosis [3], the engulfment of pathogens, the production of reactive oxygen species (ROS) [4], and degranulation [5], where they release antimicrobial proteins that directly combat pathogens [5]. Additionally, neutrophils can form neutrophil extracellular traps (NETs), web-like structures composed of DNA and antimicrobial proteins that trap and kill bacteria [6]. Together, these functions allow neutrophils to play a critical role in the initial stages of the immune response, controlling infections and triggering further inflammatory signaling [7].

The aging process is accompanied by a broad decline in immune function, a phenomenon known as immunosenescence [8], which affects both the innate and adaptive immune systems. Immunosenescence can lead to weakened immune surveillance [9], making aged people more vulnerable to infections, chronic inflammation, and various age-related diseases [10,11]. With age, immune cells, including neutrophils, undergo changes that alter their typical responses to infections and inflammatory signals [12,13]. In aged individuals, we observe a phenomenon known as inflamm-aging [14], marked by high levels of circulating pro-inflammatory cytokines in the absence of infection, combined with a slower, less effective immune response [15,16]. This change disrupts the immune homeostasis, contributing to poor outcomes in older individuals following infections [17,18].

Previous studies have shown that aging affects several functional aspects of neutrophils, resulting in distinct differences in the immune response between aged and young mice [19]. It is suggested that chemotaxis, the directed migration of neutrophils, is reduced in aged mice, as they recruit fewer neutrophils in response to inflammatory signals like Keratinocyte-derived cytokine (KC) compared to younger mice [20]. This lower leukocyte accumulation in aged mice is also associated with reduced levels of ICAM-1 in wounds at early stages [15]. Furthermore, aged neutrophils show increased baseline levels of ROS production and altered NET formation, potentially resulting in hyperactivity and excessive inflammatory responses [21,22]. Additionally, studies show that the phagocytic capacity of aged neutrophils may change [19], leading to greater phagocytic activity in inflamed tissues than in younger cells, which can potentially exacerbate inflammation and tissue damage [23]. Chronic periodontitis, marked by osteolysis and tissue damage, is characterized by a predominant infiltration of lymphocytes, particularly T and B cells, rather than neutrophils. This shift reflects a transition from an acute, neutrophil-driven response to a sustained, adaptive immune response that underlies chronic tissue inflammation and destruction [24,25]. However, neutrophils remain functionally relevant as they represent the primary initial immune responder in the mouth. Recent evidence has highlighted the functional heterogeneity and activation states of oral neutrophils in both health and disease. In chronic periodontitis, oral neutrophils exhibit significantly elevated phagocytic activity and degranulation compared to those from healthy individuals [26]. Indeed, distinct neutrophil subsets with enhanced CD63 expression and increased phagosome and granule content were found in diseased tissues. Oral neutrophils adopt a hyperactive phenotype in the inflamed periodontal environment. Similarly, previous studies showed that ligature-induced periodontitis in mice leads to upregulation of activation markers such as CD11b and CD66a on oral neutrophils, further confirming their heightened inflammatory status during periodontal disease [27]. Thus, in chronic lesions, especially in aged individuals, neutrophil-dysregulated activity could exacerbate tissue damage.

Despite the growing understanding of immunosenescence, there is limited research on how specific functions of neutrophils differ between young and aged animals, particularly in model organisms like mice [28,29]. Understanding these differences is crucial, as age-related changes in neutrophils could contribute to increased infection rates, prolonged wound healing, and chronic inflammation [30]. By studying these variations in a controlled animal model, this research could reveal important insights into age-associated immune dysfunctions. This knowledge has the potential to guide the development of therapeutic strategies aimed at enhancing immune responses in the aging population, ultimately improving health outcomes and resilience against infections in older individuals.

This study investigates how aging alters neutrophil function by comparing recruitment, activation, and inflammatory responses in young and aged mice, with the goal of understanding the impact on immune health and disease outcomes in older individuals.

## 2. Materials and Methods

### 2.1. Animals

All animal care and experimental procedures were conducted in accordance with the Guide for the Humane Use and Care of Laboratory Animals and were approved by the University of Toronto Animal Care Committee under protocol number 20012249. This study used C57BL/6J mice, a strain selected for its extensive use and well-documented background, particularly in aging and immunology studies. The cohort included 6-week-old mice (N = 8; 4 females and 4 males), referred to as “young”, corresponding to 18–20 years old humans, and 18-month-old mice (N = 8; 4 females and 4 males), classified as “aged”, corresponding to >60 years old humans, based on the National Institute on Aging (NIA) classification and previous work from our lab [31]. The mice were purchased from Jackson Laboratory and housed at the University of Toronto Animal Care Facility under a 12-h light-dark cycle at a constant temperature, with free access to food and water.

### 2.2. Isolation of Murine Bone Marrow Neutrophils for Chemotaxis

Mice were euthanized via CO_2_ inhalation. Femurs and tibias were harvested to isolate bone marrow (BM) cells. The BM cells were layered onto discontinuous Percoll gradients (80%/65%/55%; Sigma, Oakville, ON, Canada) to separate mature neutrophils. Neutrophils were collected at the 80%/65% interface, yielding a population with >90% purity and >98% viability, confirmed by Wright stain and Trypan blue exclusion, respectively [32].

### 2.3. Peritonitis Induction (IP), Peritoneal Lavage, and Blood Collection

To investigate the differing roles of neutrophils in aged and young mice, we induced peritonitis in both group young and aged, to create a localized inflammatory response that recruits neutrophils to the peritoneal cavity. The peritonitis model was used as a controlled system to assess age-related neutrophil responses, serving as a proxy for inflammatory conditions like periodontitis. Peritonitis was induced by intraperitoneal injection of 2 μL or 50 μL of pHrodo Red *Escherichia coli* BioParticles (Molecular Probes, Eugene, OR, USA) was reconstituted in 100 μL PBS and injected into the peritoneal cavity using a 1 mL syringe. After 3 h, mice were euthanized by CO_2_ inhalation, and peritoneal cells were collected by lavage with 3 mL cold PBS. Blood samples were obtained by cardiac puncture with EDTA as an anticoagulant [33].

### 2.4. Neutrophil Chemotaxis Assay

A suspension of 1 × 10^6^ bone marrow PMNs (Polymorphonuclear neutrophils) in 100 μL HBSS with 1% gelatin (Sigma-Aldrich, St. Louis, MO, USA) was placed on a 5% bovine serum albumin-coated glass coverslip (22 × 40 mm, Fisher Scientific, Waltham, MA, USA) and incubated for 10 min at 37 °C. Coverslips were inverted onto a Zigmond chamber (Neuro Probe, Gaithersburg, MD, USA), with HBSS and HBSS containing 10^−6^ M N-formyl-methionyl-leucyl-phenylalanine (fMLP; Sigma-Aldrich, St. Louis, MO, USA) added to the right and left chambers, respectively. PMN migration was recorded via time-lapse video microscopy every 20 s over 15 min using a Nikon Eclipse E1000 microscope, and cell tracking software (Retrac, v2.1.01) was used for analysis [34].

### 2.5. PMN Superoxide Production Assay

A suspension of 1 × 10^6^ BM PMNs in 100 μL PBS with 10 mM D-glucose was prepared in a 2 mL cuvette (Biomart, Toronto, ON, Canada). A total of 880 μL PiCM-G buffer (138 mM NaCl, 2.7 mM KCl, 0.6 mM CaCl_2_, 1 mM MgCl_2_, 5 mM glucose, 10 mM NaH_2_PO_4_/Na_2_HPO_4_) and 10 μL equine ferricytochrome c (Sigma-Aldrich, St. Louis, MO, USA) were added. The cuvettes were incubated at 37 °C with gentle shaking. PMNs were stimulated with either 1 μM phorbol myristate acetate (PMA) or 1 μM fMLP to achieve final concentrations of 1μM for each reagent for 30 min. Finally, the absorbance of reduced cytochrome c was measured at 550 nm with an Ultrospec 3000 UV/Visible Spectrophotometer (Pharmacia Biotech, Uppsala, Sweden).

### 2.6. Anti-Mouse Antibodies for Flow Cytometry

The following antibodies were used for flow cytometry analysis: Ly6G (1A8; BD, San Jose, CA, USA), F4/80 (BM8; Biolegend, San Diego, CA, USA), CD11b (M1/70; Biolegend), CD66a (CC1; eBioscience, San Diego, CA, USA), a-Citrullinated histone 3 (a-H3Cit; polyclonal; Abcam, Cambridge, UK), and goat anti-rabbit immunoglobulin G H&L AF488 (Cell Signaling Technology, Danvers, MA, USA).

### 2.7. Flow Cytometry

Sample acquisition was performed using an SA3800 flow cytometry analyzer. Multicolor flow cytometry panels were developed to characterize murine neutrophil surface markers, with gating on Ly6G+ CD66+ populations. Single-stained cells were used for compensation, and data were analyzed with FlowJo software (version 10, TreeStar, Ashland, OR, USA) [33].

### 2.8. Phagocytosis by Flow Cytometry

To assess phagocytic activity, peritoneal lavage fluid was recovered following 3 h of peritonitis induction using 2 µL pHrodo. The collected cells were fixed with methanol-free formaldehyde at a final concentration of 1.6% for 15 min. After fixation, the cells were resuspended in flow-assisted cell sorting (FACS) buffer, which consisted of Hank’s Balanced Salt Solution (without calcium or magnesium), 1% bovine serum albumin, and 2 mM EDTA. Approximately 500,000 cells were then blocked with 2 mg of mouse immunoglobulin G (Sigma-Aldrich, St. Louis, MO, USA) and 60–80 mg of rat serum (Sigma-Aldrich, St. Louis, MO, USA) for 20 min to minimize non-specific binding. Following blocking, the cells were labeled with Ly6G and F4/80 antibodies for 30 min on ice in the dark, after which they were washed three times with FACS buffer. The samples were then prepared for flow cytometry analysis [33].

### 2.9. NETosis and CD Marker Expression by Flow Cytometry

Neutrophil NETosis and activation marker expression were evaluated using bone marrow (BM), blood, and peritoneal lavage fluid. All samples were fixed with methanol-free formaldehyde at a final concentration of 1.6% for 15 min on ice. To eliminate red blood cells (RBCs), samples were treated twice with Pharm Lyse (BD Biosciences, San Jose, California, USA) for 5 min on ice. The cells were then resuspended in FACS buffer (Hank’s Balanced Salt Solution, 1% bovine serum albumin, 2 mM EDTA), and approximately 500,000 cells were blocked with 2 mg of mouse immunoglobulin G and 60–80 mg of rat serum for 20 min.

Following blocking, cells were sequentially labeled with primary antibodies against citrullinated histone 3 (a-H3Cit) and secondary antibodies (goat anti-rabbit AF488) before being stained with antibodies against Ly6G, F4/80, CD66a, and CD11b. The samples were subsequently prepared for flow cytometry analysis [33].

### 2.10. Ligature-Induced Alveolar Bone Loss (ABL)

The Ligature-induced ABL mimics the microbial and inflammatory environment seen in periodontitis, making it possible to study how immune responses contribute to bone resorption and tissue degradation in aged versus young mice. For the assessment of alveolar bone loss, young and aged mice were anesthetized using a mixture of 100 mg/kg body weight ketamine and 10 mg/kg body weight xylazine and positioned supine under a stereomicroscope. A 9-0 silk suture was carefully placed in the gingival sulcus around the maxillary left second molar (M2) and tied on the palatal side using a surgeon’s knot (designated as day 0) [35]. After 7 days [36], the mice were euthanized, and the presence of the ligature was verified prior to further processing [35].

The maxillae were fixed in 10% formaldehyde, skinned, and defleshed. Dry skulls were then stained with methylene blue (1% in water) for 1 min. Images of the buccal aspects of the second molars were captured at 5× magnification using a video camera (PixeLINK, Ottawa, ON, Canada) mounted on a Nikon Eclipse E1000 stereomicroscope. Mesiobuccal and distobuccal alveolar bone loss was quantified morphometrically from the cemento-enamel junction to the alveolar bone crest using ImageJ software (version 1.53t, National Institutes of Health, Bethesda, MD, USA) [31,37].

### 2.11. Statistical Analysis

Data are presented as the mean ± standard error of the mean (SEM). SEM was used instead of the standard deviation (SD) to represent the precision of the sample mean when comparing groups (young vs. aged mice). SEM highlights the reliability of the mean differences, making it more appropriate for inferring population-level effects. Statistical comparisons were conducted using a one-way analysis of variance (ANOVA) with Bonferroni’s Multiple Comparison Test for post hoc analysis or a two-way Student’s *t*-test. A *p*-value of less than 0.05 was considered statistically significant. The micrographs presented are representative of at least three separate experiments. All statistical analyses were performed using GraphPad Prism software (version 5.00) [33].

## 3. Results

### 3.1. Neutrophil Chemotaxis in Young vs. Aged Mice

**Figure 1:**
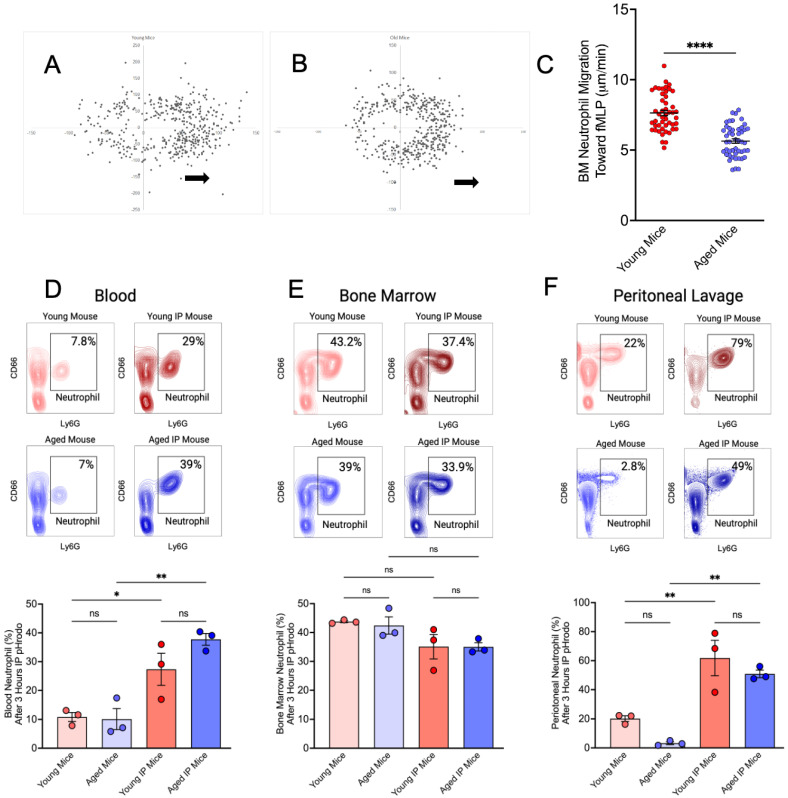
Young and aged murine neutrophil translocation in vitro and vivo. Percoll gradient centrifugation was performed, and bone marrow (BM) neutrophils were recovered. Neutrophil Chemotaxis toward fMLP was determined using a Zigmond chamber. (**A**,**B**) XY-plots represent the endpoints of migrating neutrophils with respect to the origin. Arrows indicate the fMLP gradient. (**C**) Young mice BM neutrophils migrate faster than aged mice neutrophils, each dot representing a neutrophil (Mean ± SEM N = 48). *p*-values were determined by a 2-tailed paired Student *t*-test. **** *p* ≤ 0.0001. (**D**–**F**) After 3 h of peritoneal injection of 2 µL of pHrodo Red *Escherichia coli* BioParticles (Molecular Probes) of mice, peritoneal lavage was recovered, heart blood was collected, and BM was flushed. All samples were fixed. After RBC lysis, all samples were counted and labeled for flow cytometry. The neutrophil number for each sample was obtained by the total counted cell number multiplied the % of gated neutrophil number from flow cytometry (Mean ± SEM N = 3). *p*-values were determined by ANOVA with Bonferroni’s Multiple Comparison Test * *p* ≤ 0.05, ** *p* ≤ 0.01, ns = not significant.

Previous studies have shown that reduced neutrophil chemotaxis contributes to delayed infection resolution in aged individuals [38]. To further investigate this, we evaluated neutrophil migration toward an fMLP gradient in vitro using a Zigmond chamber. Neutrophil velocity was significantly higher in young mice (7.64 ± 1.31 μm/min) compared to aged mice (5.64 ± 1.10 μm/min; Figure 1A–C). Each group was composed of 3 mice. Total neutrophil counts were quantified by combining data from flow cytometry (Ly6G+/CD66a+/F4/80-cells) and a Coulter counter. Following peritonitis induction, aged mice exhibited systemic neutrophilia, which was not observed in young mice, indicating an age-related systemic response (Figure 1D). Aged IP mice exhibited a higher neutrophil percentage compared with young IP mice, although this difference was not statistically significant. However, analysis of the absolute neutrophil count revealed a significant increase in aged IP mice relative to young IP mice (Table 1). Bone marrow neutrophil depletion was evident in both young and aged mice post-induction, suggesting neutrophil mobilization to inflammatory sites. Nevertheless, no statistically significant result was observed (Table 2, Figure 1E). In response to peritoneal pHrodo injection, young mice demonstrated greater neutrophil recruitment to the peritoneal cavity compared to aged mice, although this difference was not statistically significant. Analysis of the absolute neutrophil count revealed that this difference was statistically significant (Table 3). Additionally, a significant increase in neutrophil recruitment was observed between young vs. young IP mice and aged vs. aged IP mice (Figure 1F).

### 3.2. Neutrophil Superoxide Production in Young vs. Aged Mice

**Figure 2:**
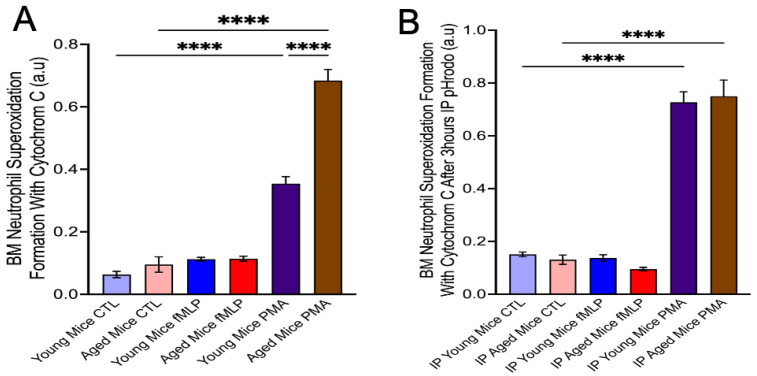
Neutrophil Superoxide Formation with Cytochrome C. Percoll gradient centrifugation was performed, and bone marrow (BM) neutrophils were recovered (**A**) in a rest situation, aged mice BM neutrophils generated higher superoxide than the young mice after stimulation with 0.1 µM PMA for 30 min. PMA induced higher superoxide formation in neutrophils than the control, and fMLP stimulated neutrophils in both young and aged mice. (Mean ± SEM N = 3) (**B**) After 3 hours’ induction of peritonitis with 50 µL pHrodo, there was no significant difference in superoxide production between young and aged mice neutrophils with the stimulation of PMA, but the ROS production of neutrophils was higher than their own control or fMLP stimulation (Mean ± SEM N = 3) *p*-values were determined by ANOVA with Bonferroni’s Multiple Comparison Test **** *p* < 0.0001. a.u: absorbance units. ROS: Reactive oxygen species. Each experiment result was from 1 million of neutrophils.

Aged mice exhibit chronic low-grade inflammation, which can influence neutrophil function. To investigate this, we measured baseline superoxide production in blood, bone marrow, and peritoneal neutrophils using cytochrome c reduction from different conditions in a group of 3 mice. Our results indicated that aged mice displayed significantly higher baseline superoxide production compared with young mice after stimulation with PMA (Figure 2A). However, after peritonitis induction with 50 μL pHrodo, no significant difference in reactive oxygen species (ROS) production was observed between young and aged mice, as 80–90% of bone marrow neutrophils in both groups were primed by the inflammatory stimulus. Furthermore, PMA-stimulated neutrophils produced substantially more ROS than non-stimulated or fMLP-stimulated cells (Figure 2B).

### 3.3. Neutrophil NETosis in Young vs. Aged Mice

**Figure 3:**
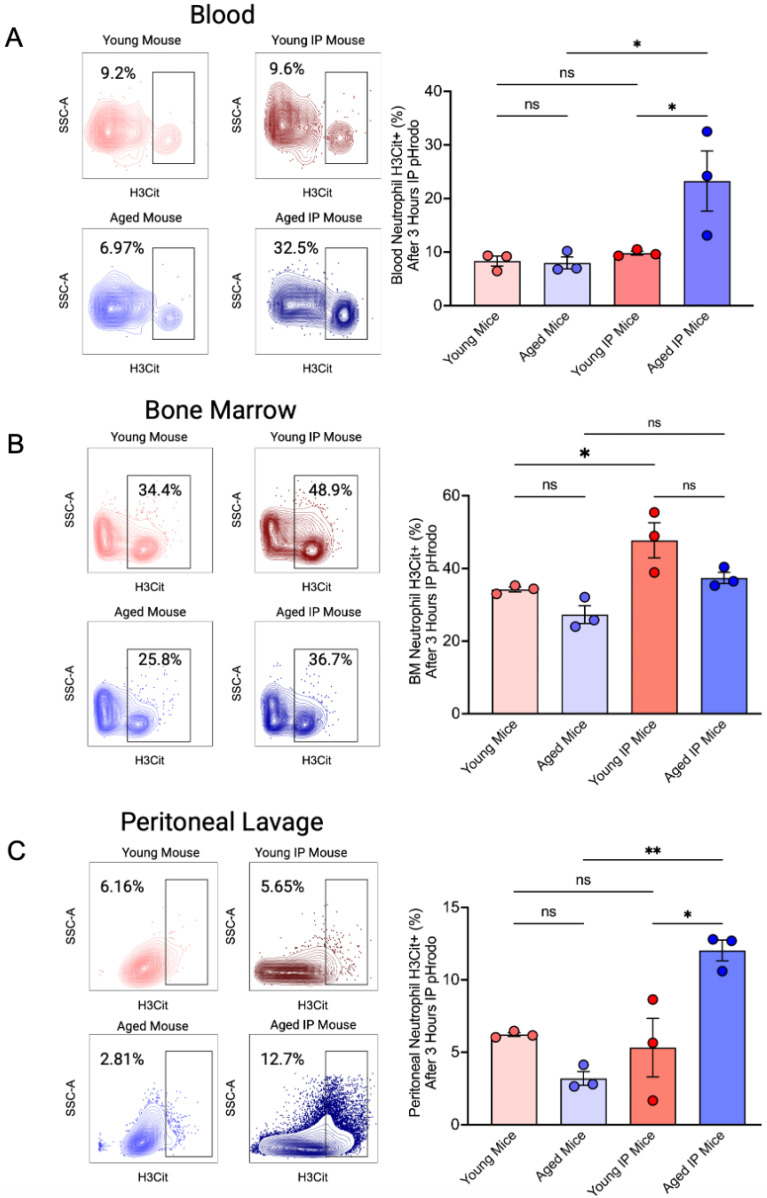
NETosis marker Histone3 Cit Expression in Neutrophils. After 3 h of peritonitis induction of mice with 2 µL pHrodo peritoneal injection, heart blood was collected, peritoneal lavage was recovered, and BM was flushed. After fixation of samples and lysis of RBC for blood samples, labeled H3Cit (AF488) for flow cytometry; (**A**) Representative flow cytometry plots showing H3Cit^+^ neutrophils in blood from young and aged mice, either untreated or injected with pHrodo: IP. Quantification shows a significant increase in NETosis (H3Cit^+^ cells) in aged IP mice. (**B**) Bone marrow neutrophils from the same groups, showing increased NETosis in young IP mice compared to young mice. (**C**) Peritoneal lavage neutrophils show increased H3Cit^+^ expression in aged IP mice, indicating enhanced local NETosis following stimulation. (Mean ± SEM N = 3); *p*-values were determined by ANOVA with Bonferroni’s Multiple Comparison Test * *p* < 0.05, ** *p* < 0.01, ns = not significant.

To evaluate NETosis, we labeled neutrophils from bone marrow, blood, and peritoneal lavage with Histone3 Cit (NETosis marker) from the 4 different groups of 3 mice. Our results indicated that blood neutrophils from aged IP mice exhibited significantly elevated Histone3 Cit (H3Cit) expression compared with aged mice and young IP mice (Figure 3A). In bone marrow neutrophils, a decreasing trend in H3Cit expression was observed following peritonitis induction in aged mice, although this change was not statistically significant. Additionally, a significantly higher percentage of H3Cit^+^ neutrophils was detected in young IP mice compared with young mice (Figure 3B). However, this difference was not observed when analyzing the absolute count of H3Cit^+^ neutrophils (Table 2). Furthermore, neutrophils from the peritoneal lavage of aged mice displayed increased NETosis after peritonitis induction compared with both aged mice and young IP mice without peritonitis induction (Figure 3C).

### 3.4. Neutrophil Phagocytosis in Young vs. Aged Mice

**Figure 4:**
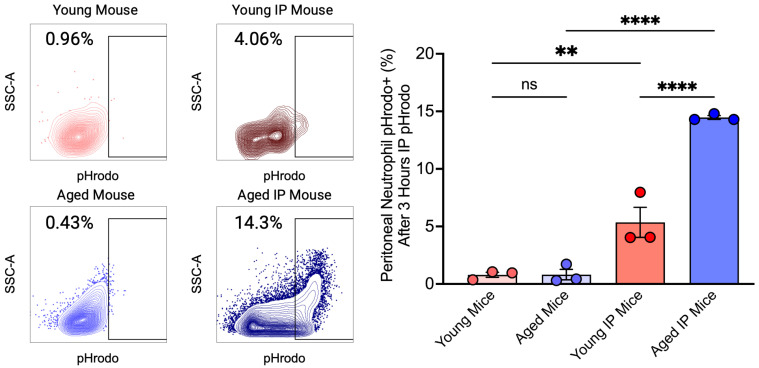
Neutrophil phagocytosis of pHrodo. After 3 h of peritoneal injection with 2 µL pHrodo, peritoneal lavage was recovered and fixed, labeled with Ly6G (1A8), and F4/80 (BM8) for flow cytometry analysis with SA3800. Aged mice seem to have a higher percentage of pHrodo-positive neutrophils than young mice. Mean ± SEM N = 3, *p*-values were determined by ANOVA with Bonferroni’s Multiple Comparison Test, ** *p* < 0.01, **** *p* < 0.0001, ns = not significant.

To investigate potential age-related differences in neutrophil phagocytosis in response to acute inflammation, we analyzed phagocytic neutrophils recovered from peritoneal lavage three hours after pHrodo administration of 3 mice per group. Flow cytometry analysis revealed that aged IP mice exhibited a significantly higher percentage of phagocytic neutrophils compared with both aged mice and young IP mice. Although an increase in phagocytic activity was observed in young IP mice compared with young mice, the significantly higher activity in aged IP mice relative to young IP mice suggests enhanced phagocytic capacity in aged mice (Figure 4).

### 3.5. Age-Related Alveolar Bone Loss in Ligature-Induced Periodontitis

**Figure 5:**
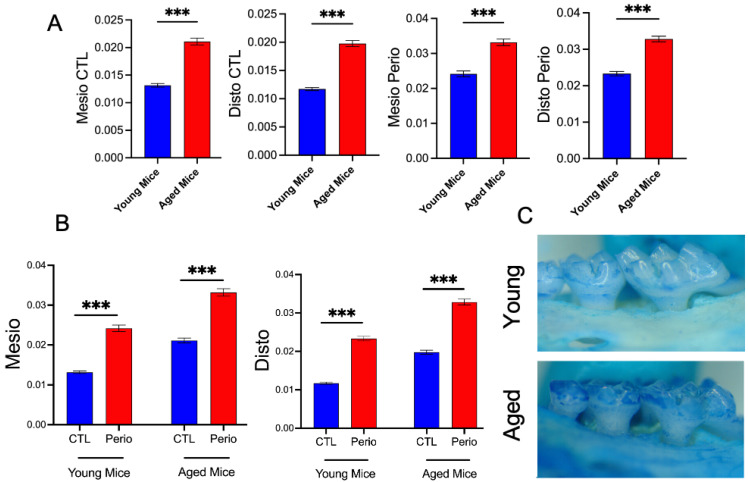
Mesiobuccal and Distobuccal Bone Loss. After ligature placement for 7 days, Maxillae were harvested and fixed with 10% formaldehyde. Dry skulls were stained with methylene blue (1% in water) for 1 min. Images of the buccal aspects of second molars were taken in X5 magnification using a video camera (PixeLINK, Ottawa, ON, Canada) mounted onto a stereomicroscope (Nikon Eclipse E1000). Mesiobuccal and Distobuccal alveolar bone loss was measured by morphometry from the cemento-enamel junction to the alveolar bone crest with ImageJ software. (**A**) Quantification of bone loss at the mesial and distal sites of molars in control and ligature-induced periodontitis (Perio) models in young and aged mice. Aged mice exhibit significantly greater alveolar bone loss in both control and periodontal conditions. (**B**) Comparison of mesial and distal bone loss between control (CTL) and periodontal conditions in both age groups confirms that aged mice experience more severe bone loss upon periodontal challenge. (**C**) Representative images of alveolar bone stained with methylene blue from young and aged mice, illustrating more pronounced bone resorption in aged samples. (Mean ± SEM N = 8), *p*-values were determined by ANOVA with Bonferroni’s Multiple Comparison Test *** *p* < 0.001.

To investigate the impact of aging on periodontal tissue degradation in periodontitis, we employed a ligature-induced periodontitis model was performed on 8 mice per group. Following seven days of ligature placement, aged mice exhibited significantly greater alveolar bone loss compared with young mice, as measured at both the mesiobuccal and distobuccal sites of the second molars (Figure 5A,B). This heightened bone loss in aged mice was consistently observed across both regions, indicating an age-related susceptibility to periodontal tissue degradation (Figure 5C). Altogether, these findings demonstrate that older mice are more prone to bone loss than their younger counterparts, highlighting the exacerbating effect of aging on periodontal breakdown.

## 4. Discussion

**Figure 6:**
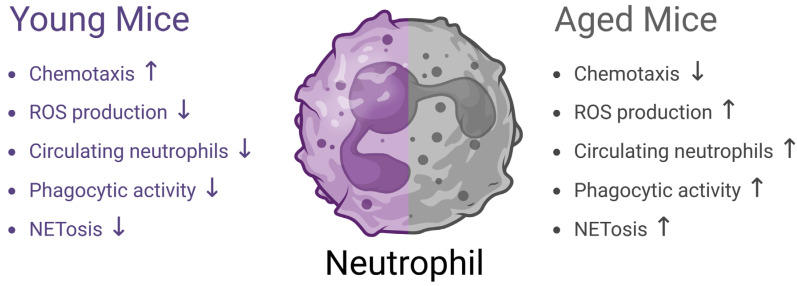
Young vs. aged mice neutrophil impact on periodontal health. Schematic representation of young vs. aged mice neutrophil function. ↑ indicates an increase in the parameter, while ↓ indicates a decrease.

This study provides new insights into the distinct functional responses of neutrophils in aged versus young mice during acute infection. Both mouse and human periodontitis models have shown that oral inflammatory disease can alter peripheral neutrophil counts and enhance their priming potential, leading to a hyperinflammatory neutrophil response when a second, unrelated inflammatory trigger is present. [39] To further explore this systemic dimension of neutrophil dysregulation, we employed the peritonitis model as a complementary approach. Although peritonitis and periodontitis are anatomically distinct, both involve PMN-driven innate immune responses, and the peritonitis model allows for controlled, acute evaluation of neutrophil recruitment, activation, and function. This model enabled us to determine whether aging-related neutrophil hyperreactivity observed in periodontitis is localized to the oral environment or reflects a broader systemic inflammatory phenotype. The findings support the concept of converging inflammatory signals from two distinct sites—oral and peritoneal—promoting synergistic PMN-mediated hyperinflammatory responses. This interaction underscores how chronic inflammatory diseases like periodontitis may sensitize the immune system, amplifying responses to secondary inflammatory challenges. Our findings demonstrate that aged mice exhibit impaired directional migration of bone marrow neutrophils toward fMLP stimulation in vitro, reduced neutrophil recruitment to the peritoneal cavity, and elevated systemic neutrophilia following peritonitis induction (Figure 1 and Figure 6). These observations are consistent with previous reports indicating that age-associated declines in neutrophil chemotaxis contribute to delayed infection resolution in older animals [15,23]. Similar age-related neutrophil dysfunction has been documented in other inflammatory models. For example, in severe *C. difficile* infection, aged mice display a diminished granulocyte response in the intestine, accompanied by elevated circulating white blood cells, granulocytes, and IL-17A levels [15]. Likewise, zymosan-induced peritonitis in aged animals results in a blunted local neutrophil response but paradoxically promotes fresh neutrophil migration [7]. In contrast, burn trauma models reveal significantly increased lung endothelial ICAM-1 expression in aged mice, correlating with prolonged pulmonary neutrophilia [40]. Following *Streptococcus pneumoniae* infection, aged mice also exhibit exacerbated inflammation due to reduced IL-10 production and increased chemokine release [41]. Moreover, aging has been shown to alter immune responses in ischemic stroke, with older mice displaying heightened neutrophilia and increased neutrophil infiltration into brain tissue [22]. The variability in these outcomes likely reflects differences in injury models and the unique microenvironmental factors of each affected organ.

The concept of “inflamm-aging” describes the low-level, chronic inflammatory stress associated with aging [22,42]. In older mice, this chronic inflammatory state leads to widespread immune cell priming, characterized by elevated inflammatory marker expression across multiple tissues [16]. ROS production from dysfunctional mitochondria can then further exacerbate inflammatory responses by activating the NF-κB signaling pathway [43]. In our study, aged bone marrow neutrophils exhibited significantly higher baseline ROS production following PMA stimulation despite a reduced capacity for dynamic ROS generation after peritonitis induction (Figure 2 and Figure 6). These findings are consistent with previous reports demonstrating that aged neutrophils produce higher baseline reactive species levels compared to younger counterparts [22]. Similar trends have been observed in models of ischemic stroke, where aged mice not only show increased mortality and morbidity but also display elevated ROS production by neutrophils, contributing to greater tissue damage and poor recovery [44]. The cumulative rise in oxidative stress and cellular damage with age is thought to underlie multiple age-related pathologies, including cognitive decline [45] and periodontal disease [46].

Although inflammation is essential for host defense, chronic inflammation in aging—largely driven by sustained neutrophil activation—can become maladaptive [42]. This resulted in an exaggerated inflammatory reaction, with increased NETosis and phagocytic activity, amplifying tissue damage (Figure 3 and Figure 6). Prior studies have reported similar findings, with aged neutrophils exhibiting hyperinflammatory responses, while interventions such as bone marrow rejuvenation have been shown to partially reverse this age-associated dysregulation [22]. Additionally, our data revealed that aged neutrophils displayed significantly higher phagocytic activity compared to their younger counterparts (Figure 4 and Figure 6), suggesting that age-related immune priming enhances phagocytosis during inflammation [47].

To further explore the impact of aging on periodontal tissue degradation, we employed a ligature-induced periodontitis model. This approach allowed us to simulate the effects of subgingival biofilm accumulation, promoting localized inflammation and alveolar bone loss [31,37]. Our results demonstrated that highly primed neutrophils, characterized by robust phagocytosis and NETosis, infiltrated inflamed periodontal tissues in aged mice (Figure 3, Figure 5 and Figure 6). We suggest that this excessive neutrophil activity contributed to an imbalance between pro-oxidant and antioxidant levels [48], further promoting tissue destruction. Additionally, reduced immune regulation [20] and increased cellular senescence in aged mice [49] likely exacerbated the periodontal damage, resulting in significantly greater alveolar bone loss compared with younger mice (Figure 5). These findings are in line with recent studies indicating that aging intensifies periodontal tissue degradation by enhancing both local inflammation and systemic immune dysregulation [50,51]. Moreover, emerging evidence suggests that age-related gut microbiota and metabolic imbalances may further promote bone loss, highlighting the potential of microbiome-targeted therapies for mitigating periodontal disease in older individuals [52]. Indeed, age-related changes in the oral microbiome significantly influence host–microbe interactions and may contribute to the pathogenesis of inflammatory conditions in older individuals [53]. With aging, the composition and diversity of the oral microbiota undergo shifts, often characterized by an increase in pathogenic species and a decrease in beneficial commensals [54]. This microbial dysbiosis not only reflects age-related ecological pressures but also plays an active role in modulating immune responses. An imbalanced microbiome may prime neutrophils for a hyperactive state, leading to exaggerated inflammatory responses [55]. The resulting neutrophil dysfunction—marked by increased ROS production, delayed apoptosis, and heightened degranulation—can create a feed-forward loop of tissue damage and microbial imbalance [56]. This vicious cycle of dysbiosis and immune dysregulation may serve as a critical co-factor in promoting chronic inflammation and alveolar bone loss in the aging population, underscoring the need to consider host–microbiome interactions as a therapeutic target in age-associated periodontal disease. Notably, age-related changes in the oral microbiome have been shown to significantly influence host–microbe interactions, potentially exacerbating immune dysregulation and contributing to the pathogenesis of inflammatory conditions such as periodontitis in older individuals [57]. These findings highlight the complex interplay between immunosenescence and microbial shifts, emphasizing the importance of age as a factor in periodontal disease progression. Thus, targeting these altered host–microbiome dynamics may open new avenues for precision therapies aimed at mitigating periodontal breakdown and preserving oral health in the elderly. Although we demonstrated that aged mice experience greater alveolar bone loss in the ligature-induced model (Figure 5), the precise cellular and molecular mechanisms underlying this disparity remain unclear. While this study demonstrates that neutrophil dysfunction and immune dysregulation coincide with increased periodontal tissue damage in aged mice, further investigation is needed to fully understand the underlying mechanisms. Indeed, it remains unclear how directly these changes modulate clinical outcomes in elderly individuals. Although neutrophil hyperactivity may promote inflammatory responses, further studies are required to determine its impact in the clinical setting. This translational gap underscores the need for longitudinal and mechanistic studies in human populations to validate these findings and evaluate their relevance in guiding therapeutic strategies. Moreover, future research should assess cytokine profiles, immune cell interactions, and local inflammatory signaling in aged periodontal tissues. The limited sample size and the use of a ligature-induced model—which does not fully reflect the chronic, polymicrobial complexity of human periodontitis—represent key areas for improvement in future studies. Nonetheless, these findings underscore the need to explore targeted therapies that modulate neutrophil hyperactivity, such as by reducing oxidative stress or NET formation. In the context of a rapidly aging global population, such approaches hold promise for improving periodontal care and guiding immune-based interventions in 21st-century dentistry.

## 5. Conclusions

Our study highlights clear functional disparities in neutrophil responses between young and aged mice during acute inflammation.

Aged neutrophils exhibit heightened phagocytic activity and increased NETosis.

These changes are accompanied by excessive ROS production and elevated inflammatory byproducts.

Together, these factors contribute to greater tissue damage in aged mice.

These findings underscore the complexity of neutrophil dysfunction with aging and highlight the need for targeted therapeutic strategies to mitigate age-related inflammatory damage and protect both periodontal and systemic health.

## Figures and Tables

**Table 1:** Absolute count of neutrophils and H3cit^+^ neutrophil in blood.

Group (Blood/100 μL)	Neutrophil (Mean ± SD)	H3cit^+^ Neutrophil (Mean ± SD)
Young	0.8 ± 0.2 × 10^3^/µL	0.0667 ± 0.04 × 10^3^/µL
Aged	0.99 ± 0.4 × 10^3^/µL	0.1863 ± 0.04 × 10^3^/µL
Young IP	1.9 ± 0.4 × 10^3^/µL	0.0790 ± 0.03 × 10^3^/µL
Aged IP	3.19 ± 0.2 × 10^3^/µL	0.7422 ± 0.04 × 10^3^/µL
**Comparison**	**Neutrophil (*p*-value)**	**H3cit^+^ Neutrophil (*p*-value)**
Young vs. Aged	ns	ns
Young vs. Young IP	0.0118 *	<0.005 ***
Young vs. Aged IP	<0.0001 ****	ns
Aged vs. Young IP	0.03 *	0.0025 **
Aged vs. Aged IP	0.0001 ****	ns
Young IP vs. Aged IP	0.0048 **	ns

Data are presented as mean ± SEM. Statistical significance is indicated as follows: *p* < 0.05 (*), *p* < 0.01 (**), *p* < 0.001 (***), *p* < 0.0001 (****); ns = not significant.

**Table 2:** Absolute count of Neutrophil and H3cit^+^ Neutrophil in BM.

Group (BM/250 μL)	Neutrophil (Mean ± SD)	H3cit^+^ Neutrophil (Mean ± SD)
Young	17.552 ± 5.16 × 10^3^/µL	6.048 ± 1.96 × 10^3^/µL
Aged	17.32 ± 2.92 × 10^3^/µL	4.788 ± 1.488 × 10^3^/µL
Young IP	9.652 ± 3.8 × 10^3^/µL	4.5 ± 1.54 × 10^3^/µL
Aged IP	13.792 ± 2.48 × 10^3^/µL	5.164 ± 1.0 × 10^3^/µL
**Comparison**	**Neutrophil (*p*-value)**	**H3cit^+^ Neutrophil (*p*-value)**
Young vs. Aged	ns	ns
Young vs. Young IP	ns	ns
Young vs. Aged IP	ns	ns
Aged vs. Young IP	ns	ns
Aged vs. Aged IP	ns	ns
Young IP vs. Aged IP	ns	ns

Data are presented as mean ± SEM. Statistical significance is indicated as follows: ns = not significant.

**Table 3:** Absolute count of neutrophils, H3cit^+^ neutrophil, and pHrodo^+^ neutrophil in peritoneal.

Group (Peritoneal/2 mL)	Neutrophil (Mean ± SD)	H3cit^+^ Neutrophil (Mean ± SD)	pHrodo^+^ Neutrophil (Mean ± SD)
Young	0.2 ± 0.075 × 10^3^/µL	0.013 ± 0.004 × 10^3^/µL	0.0015 ± 0.0005 × 10^3^/µL
Aged	0.173 ± 0.06 × 10^3^/µL	0.006 ± 0.004 × 10^3^/µL	0.0015 ± 0.0020 × 10^3^/µL
Young IP	0.734 ± 0.26 × 10^3^/µL	0.064 ± 0.059 × 10^3^/µL	0.0385 ± 0.0170 × 10^3^/µL
Aged IP	0.972 ± 0.27 × 10^3^/µL	0.115 ± 0.019 × 10^3^/µL	0.1405 ± 0.0375 × 10^3^/µL
**Comparison**	**Neutrophil** **(*p*-value)**	**H3cit^+^ Neutrophil (*p*-value)**	**pHrodo^+^ Neutrophil (*p*-value)**
Young vs. Aged	ns	ns	ns
Young vs. Young IP	0.03 *	ns	ns
Young vs. Aged IP	0.005 **	0.015 *	0.0002 ***
Aged vs. Young IP	0.029 *	ns	ns
Aged vs. Aged IP	0.004 **	0.011 *	0.0002 ***
Young IP vs. Aged IP	ns	ns	0.001 **

Data are presented as mean ± SEM. Statistical significance is indicated as follows: *p* < 0.05 (*), *p* < 0.01 (**), *p* < 0.001 (***), ns = not significant.

## Data Availability

The original contributions presented in this study are included in the article. Further inquiries can be directed to the corresponding author. You can reach them by email at michael.glogauer@dentistry.utoronto.ca or by mail at 124 Edward St, Toronto, ON M5G 2L3.
